# Screening for potential metal hyperaccumulator plants in Angola: a herbarium-based approach

**DOI:** 10.1007/s10661-026-15744-w

**Published:** 2026-08-01

**Authors:** Tom Morgenstern, Christin Baumgärtel, Thea Lautenschläger, Linda Götzke, Christoph Neinhuis, Jan J. Weigand

**Affiliations:** 1 Botanical Garden, Universität Hamburg, Hamburg, Germany; 2 Institute of Botany, Technische Universität Dresden, Dresden, Germany; 3 Chair of Inorganic Molecular Chemistry, Technische Universität Dresden, Dresden, Germany; 4 Department of Chemistry and Polymer Science, Stellenbosch University, Stellenbosch, 7600 South Africa; 5 Chair of General Microbiology, Technische Universität Dresden, Dresden, Germany

**Keywords:** Metal hyperaccumulators, Herbarium-based screening, Aluminum hyperaccumulation, Manganese hyperaccumulation, Tropical soils, Angola

## Abstract

Metal hyperaccumulator plants play a key role in understanding plant adaptation to metal-rich soils and offer potential for phytoremediation and phytomining. Angola hosts highly weathered tropical soils, yet its flora remains unexplored, mainly with respect to metal accumulation traits. This study presents the first broad, herbarium-based screening of Angolan plant specimens to identify potential hyperaccumulator taxa and evaluate taxonomic and spatial patterns of metal accumulation. A total of 234 samples from 132 species were analyzed for aluminum, manganese, nickel, copper, iron, cobalt, chromium, cadmium, lead, and zinc using microwave digestion followed by optical emission spectrometry. We identified numerous aluminum hyperaccumulators, particularly within the genus *Landolphia,* with contents in leaves up to 4.5% of dry mass, and detected extreme manganese accumulation in *Landolphia congolensis*, exceeding 2% of dry mass. These metal contents markedly exceed previously reported values for most Angolan species and reveal metal accumulation in taxa not previously recognized as hyperaccumulators. We also show strong site dependence of accumulation and consistent differences between plant organs, with leaves generally containing higher metal contents than fruits and inflorescences. Our findings significantly expand the known diversity of African metal hyperaccumulators, demonstrate the high value of herbarium collections for environmental monitoring, and provide a foundation for future work on phytoremediation potential, plant-soil interactions, and implications for human and ecosystem health in Angola.

**Supplementary Information:**

The online version contains supplementary material available at 10.1007/s10661-026-15744-w.

## Introduction

Angola exhibits a wide range of soil types, particularly in the eastern regions and along the coastline. According to the World Reference Base (WRB) for Soil Resources (Deckers et al., 1998), 17 reference soil groups occur in Angola, with inland areas largely dominated by Ferralsols and Arenosols. Arenosols alone cover more than 50% of the country’s land surface (Huntley, 2019), representing highly weathered, porous, and leached soils with low clay content. These soils typically contain low contents of most metals, although stable aluminum and iron compounds derived from the underlying parent material are abundant (Joint Research Centre of the European Commission, 2013). Low contents of essential plant nutrients such as phosphorus (P), calcium (Ca), and magnesium (Mg), combined with elevated contents of soluble and potentially toxic elements such as aluminum (Al) and manganese (Mn), strongly limit crop productivity and contribute to Angola’s overall low agricultural yield (FAO, 2015; Mascher, 1995; McCann, 2005).

Despite these challenging edaphic conditions, many native plant species have evolved adaptive strategies enabling survival and growth in metal-rich, acidic soils (Rascio & Navari-Izzo, 2011; van der Ent et al., 2013). These adaptations include mechanisms for metal exclusion, internal detoxification, and, in some cases, active accumulation of metals such as Al and Mn, as well as trace elements including nickel (Ni), cobalt (Co), and copper (Cu), which are often associated with tropical weathered soils (Jansen et al., 2002; Metali et al., 2012). Of particular interest are metal-tolerant or metal-accumulating plants, especially hyperaccumulators, which can concentrate extraordinarily high levels of specific metals in their tissues without showing toxicity symptoms (Brooks et al., 1977; van der Ent et al., 2013).

Metal hyperaccumulators are of considerable interest due to their potential applications in phytoremediation of contaminated soils, phytomining of economically valuable metals, and ecosystem restoration (Reeves et al., 2018; van der Ent et al., 2013). Beyond these applications, the accumulation of nutritionally relevant or toxic elements in wild or traditionally used plants has important implications for food security and human health (Alloway, 2013). This is especially relevant in rural regions where locally harvested or cultivated plant species constitute a substantial proportion of the diet and income.

In tropical acidic soils, aluminum and manganese accumulation is widespread and has been reported across several plant families, including Apocynaceae, Rubiaceae, Melastomataceae, and Euphorbiaceae (Jansen et al., 2002; Mesjasz-Przybylowicz et al., 2016; Metali et al., 2012). Nickel hyperaccumulation, in contrast, is taxonomically more restricted and is particularly associated with members of the Phyllanthaceae, such as *Antidesma montis-silam* and *Phyllanthus balgooyi* (Mesjasz-Przybylowicz et al., 2016; Nkrumah et al., 2018; Reeves et al., 2018). Within Apocynaceae, several genera are known aluminum accumulators, and the widespread African genus *Landolphia* has been highlighted as a potentially essential but insufficiently studied group with respect to aluminum accumulation in acidic soils (Baumgärtel et al., 2022, 2023; Jansen et al., 2002; Mesjasz-Przybylowicz et al., 2016).

The identification and study of hyperaccumulator species are especially pertinent in regions with naturally metal-rich soils or anthropogenic contamination from activities such as mining. The neighboring Democratic Republic of the Congo has yielded a high number of documented hyperaccumulators (Reeves et al., 2018), suggesting a high likelihood that similar species occur in Angola.

Monitoring the accumulation of trace and potentially toxic elements in wild and traditionally used plant species is essential for assessing environmental contamination, understanding plant-soil interactions, and evaluating potential dietary exposure risks. Previous work in northern Angola has revealed substantial variation in the elemental composition of edible plants, with metal contents ranging from nutritionally beneficial to potentially concerning levels, particularly for aluminum and manganese (Baumgärtel et al., 2023). These findings emphasize the need for taxonomically broad screening approaches that can identify species with unusual accumulation patterns across diverse landscapes.

An extensive collection of Angolan plant specimens is preserved at the Herbarium Dresdense (DR). This collection presents a valuable opportunity for large-scale elemental screening, comprising approximately 3,750 voucher specimens collected mainly since 2012 in the northern provinces of Cuanza Norte and Uíge (JACQ Herbarium, accessed 2026–01–06). The additional Herbarium Hamburgense (HBG) holds at least 4,350 Angolan specimens, most collected since 2011 in the southern part of the country. Comparable herbarium-based studies have successfully identified metal hyperaccumulator species and assessed elemental accumulation patterns in preserved plant material elsewhere (Belloeil et al., 2021; Samojedny et al., 2023; van der Ent et al., 2019), demonstrating the suitability of herbarium collections for environmental monitoring and assessment.

This study aims to apply a herbarium-based analytical approach to identify Angolan plant taxa exhibiting elevated contents of selected metals and to explore taxonomic and spatial patterns of metal accumulation. By linking plant elemental composition with ecological context, this research seeks to improve understanding of plant adaptation to metal-rich tropical soils and to contribute to sustainable land-use planning, environmental monitoring, and human health risk assessment in Angola.

## Materials and methods

**Fig. 1:**
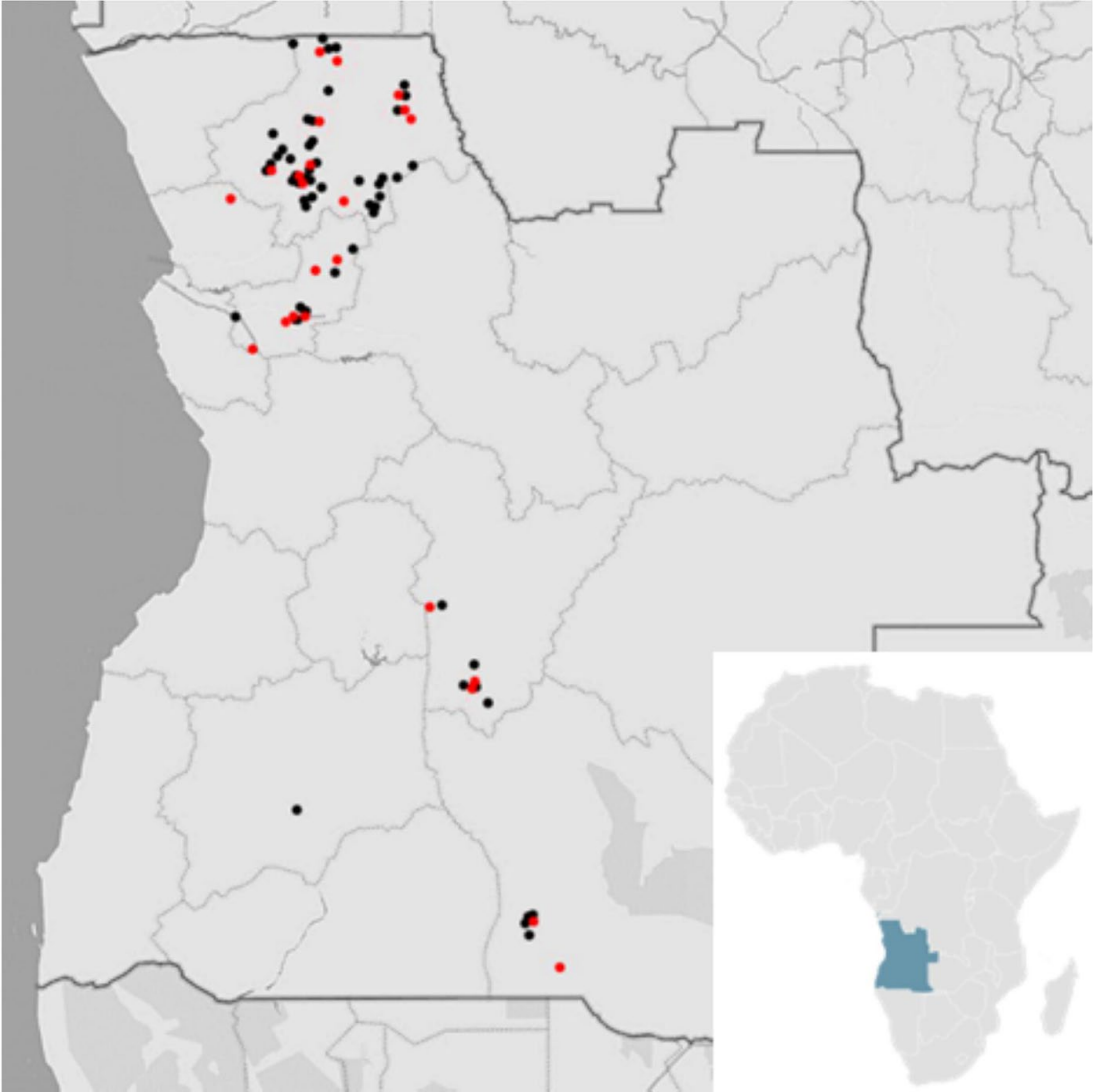
Map of Angola showing the locations of the collected herbarium specimens. Data points mark the collection locations of the herbarium specimens; red dots mark the location of specimens identified as hyperaccumulators (Data and rendering from Google Maps. Map data ©2025 Google, GeoBasis-DE/BKG (©2025))

A total of 234 samples from 132 plant species, from 59 genera, and from 31 families were selected from the Angola collection of the Herbarium Dresdense (124 species), supplemented with partly overlapping and additional species from the Herbarium Hamburgense (25 species). All samples were collected since 2011, in the provinces of Bengo, Bié, Cuando Cubango, Cuanza Norte, Huíla, and Uíge (Fig. 1). Selection was based on the *Global Hyperaccumulator Database* of the Centre for Mined Land Rehabilitation (University of Queensland), focusing on genera known to include hyperaccumulator species. Herbarium specimens without visible contamination and with sufficient plant material were preferred. Leaf material was prioritized due to its lower risk of contamination by adhering soil particles. Evaluation of samples and possible contamination was done by eye, samples with visible contamination were not used, microscopical examination was not performed. However, some of the samples showed elevated iron contents which can be a sign for adhering soil or dust particles. Potential hyperaccumulators with iron contents above 1,000 mg/kg are marked (Table 2). Due to the nature of herbarium specimens, there were no information available on the age of the plant itself or the leaves and the position of the leafs on the plant. In selected cases, stems, flowers, fruits, or tubers were also sampled (Table S1). Often times the amount and position from which the samples were taken off the herbarium specimen were dictated by the attempt to destroy the nature of the specimen as little as possible. The same principle was followed for species with more than one specimen and was decided individually for each to preserve the specimens to the best possible extend. Some species were sampled from only one location; others, when possible, were sampled from various places. A detailed list of the sampled plant organs for each species is to be found in the supplement (Supplement 1). Sample masses ranged between 21.5 mg and 910.6 mg, and each sample was processed in duplicate.

Each herbarium specimen subsample was digested and measured in duplicate (independent digestions) to assess analytical precision. Samples were digested using a microwave-assisted protocol (MARS 6, CEM GmbH) in sealed Teflon vessels. Each sample was treated with 10 mL of nitric acid (Suprapur, Carl Roth, 69%) and pre-digested for 60 min at room temperature followed by microwave digestion at 210 °C (heat ramp 20 min, 15-min holding time). After cooling, the digested samples were transferred into 50 mL volumetric flasks, filled with ultra-pure Milli-Q grade water (Millipore Corp.), and stored in 50 mL centrifuge tubes at 8 °C until analysis. After the digestion step 59 out of 234 analyzed samples showed a white precipitate. These are probably silicates which haven’t been dissolved by the acid and might have an influence on the measured metal contents in a way that the measured values might be lower than the actual content in the plants. The specific samples which showed the precipitate are marked in the table in the supplement (Table S1).

Analysis of the 10 elements—aluminum, cadmium, cobalt, chromium, copper, iron, manganese, nickel, lead, and zinc—was performed using inductively coupled plasma optical emission spectrometry (ICP-OES; Agilent 5900). External calibration was conducted using a multi-element standard solution (Perkin Elmer Pure VI, 1,000 mg/l), diluted to concentrations of 1, 5, 10, and 20 mg/l. For selected samples with high metal contents exceeding the calibration range, the standard additions method was used to minimize matrix effects. Measurements were conducted at two characteristic wavelengths per element (first wavelength used for final quantification, second wavelength as control): aluminum (396.152 nm; 237.312 nm), cadmium (214.439 nm; 226.502 nm), cobalt (238.892 nm; 228.615 nm), chromium (205.560 nm; 267.716 nm), copper (324.754 nm; 327.395), iron (259.940 nm; 238.204 nm), manganese (259.372 nm; 257.610 nm), nickel (231.604 nm; 216.555 nm), lead (182.143 nm; 220.353 nm), and zinc (213.857 nm; 202.548 nm). Instrumental measurements were performed in triplicate per solution and averaged; the corresponding relative standard deviation (RSD) values were monitored and were within normal limits (< 3% for solutions with relevant concentrations). Consistent quality of the determination was ensured by measurements of blank samples after every 20 measurements and quality controls. Because herbarium material is heterogeneous the relative percentage difference (RPD) for the duplicate digested solutions are rather high for some of the samples. Metal concentrations determined were evaluated against element-specific limits of detection (LOD) and quantification (LOQ). The LOD corresponds to three times, and the LOQ to nine times, the standard deviation of the blank measurement for the respective element. As RSD typically increases near the LOQ, values below LOQ were not interpreted quantitatively. The LOD and LOQ values are different for each plant sample and duplicate, depending on the amount of sample material weighed as well as for each element analyzed. This results in limits specific to each sample for the final mass fractions reported. However, these values are many times lower than the threshold for hyperaccumulation so that potential hyperaccumulators will not be missed because of too high LOD/LOQ. The final metal contents were calculated relative to the dry mass of the samples. Where duplicate results straddled an element-specific hyperaccumulator threshold, samples were conservatively treated as “potential” hyperaccumulators unless both duplicate digestions exceeded the threshold (see Supplement).

The threshold values at which plants are classified as hyperaccumulators vary depending on the specific element and the contents considered typical or average for non-accumulating plants. For many, but not all, elements, the scientific literature provides generally accepted data on the contents at which hyperaccumulation occurs. The following list of threshold values for this classification was compiled from a comparison of various studies on this topic (Table 1).

**Table 1:** Limit values for hyperaccumulators

	metal contents in mg/kg
Aluminum (Al)	1,000–3,000 ^[1]^
Cadmium (Cd)	100 ^[2, 3]^
Cobalt (Co)	300 ^[2]^ – 1,000 ^[4, 5]^
Chromium (Cr)	300 ^[2]^ – 1,000 ^[5]^
Copper (Cu)	300 ^[2]^ – 1,000 ^[4, 5]^
Iron (Fe)	> 10,000
Manganese (Mn)	10,000 ^[2, 5]^
Nickel (Ni)	1,000 ^[2, 6]^
Lead (Pb)	1,000 ^[2, 4, 5]^
Zinc (Zn)	3,000 ^[7]^ – 10,000 ^[5, 8]^

Standard metal contents above which plants are classified as hyperaccumulators for the respective element, values in mg/kg based on dry mass. Some threshold values differ between publications; these cases are given as a value range above which plants are considered as hyperaccumulators for that element. Literature for limit values: [1] Jansen et al., 2002; [2] van der Ent et al., 2013; [3] Krämer, 2000; [4] Malaisse et al., 1979; [5] Baker & Brooks, 1989; [6] Brooks et al., 1977; [7] Krämer, 2010; [8] Reeves & Baker, 2000.

The elements aluminum and iron are considered separately here, as they are not among the classic trace elements of hyperaccumulators. Nevertheless, publications on aluminum hyperaccumulators have been published, and threshold values have been proposed (Alloway, 2013; Belloeil et al., 2021). Since iron is one of the most abundant elements in soils, no general threshold value above which plants are considered hyperaccumulators is known. For this screening, we initially set this value at > 10,000 mg/kg.

## Results and discussion

**Fig. 2:**
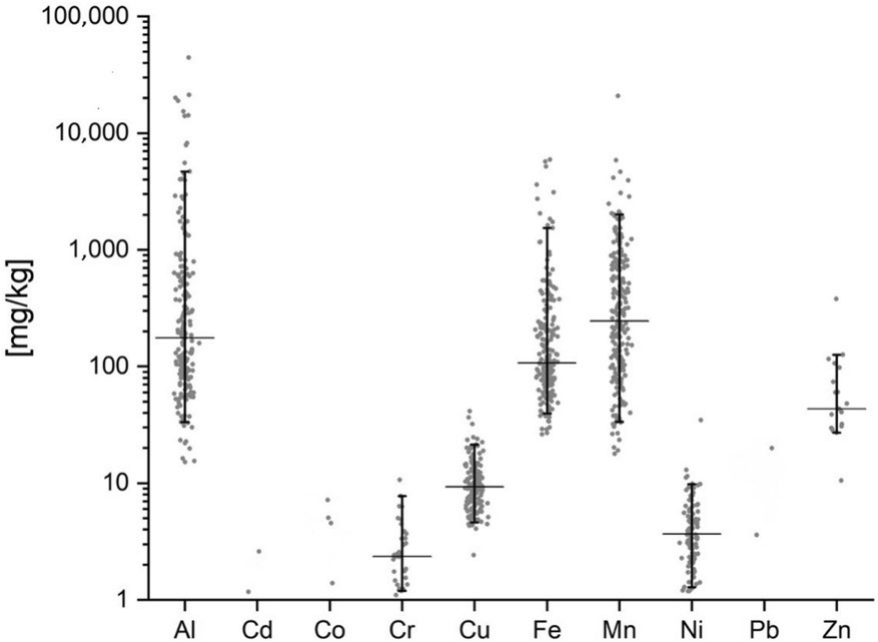
Distribution of the measured elemental contents of all plant samples. Diagram of the quantifiable element contents (above LOQ) of all plant samples, sorted by elements with median and percentiles (5–95%) in [mg/kg] based on dry mass, plotted logarithmically. Individual data points indicate mean values of duplicate determinations

Across all 234 tested samples, which showed metal contents above the LOQ, the highest median metal content was found for manganese (244.4 mg/kg), followed by aluminum (175.7 mg/kg) and iron (107.0 mg/kg), with lower values for copper and nickel. For cadmium, cobalt, chromium, lead, and zinc, the contents in most samples were below the respective LOD or LOQ (Fig. 2). The 5–95% percentiles normally illustrate typical metal content ranges of each element for non-hyperaccumulator plants. Since the selection of plants for this study is based on species (and genera with species) known to be hyperaccumulators in the hyperaccumulator database, the shown 5–95% percentiles might not be representative of typical ranges. Unless stated otherwise, all values are based on the mean of duplicate measurements. A complete overview of elemental contents for all analyzed samples is provided in the supplement (Supplement 1).

### Aluminum and Manganese hyperaccumulators

The median aluminum contents of all measured samples above LOQ is 175.7 mg/kg, and the mean is 1,291.9 mg/kg. 199 of the 234 samples tested had aluminum contents below 1,000 mg/kg. 19 samples were measured with an aluminum content between 1,000 and 3,000 mg/kg, 9 samples between 3,000 and 10,000 mg/kg, and seven samples with an aluminum content above 10,000 mg/kg (Table 2). Some samples of *Anisophyllea quangensis*, *Commelina africana*, *Landolphia camptoloba*, *Landolphia congolensis*, *Landolphia jumellei*, and *Landolphia lanceolata* exhibit particularly high aluminum contents, exceeding 1% of the dry mass. Samples of *Anisophyllea quangensis* and *Landolphia camptoloba* contained aluminum of over 2%, and one *Landolphia camptoloba* sample contained aluminum of up to 4.5% of the dry mass. In the genus Spermacoce, hyperaccumulators with low to medium aluminum contents were identified in all four species examined. The genera *Landolphia* and *Spermacoce* are particularly conspicuous. Hyperaccumulators were identified in five of the eight *Landolphia* species examined. These are also the species with the highest measured aluminum contents. In the genus *Spermacoce*, hyperaccumulators with low to medium aluminum contents were identified in all four species examined.

**Table 2:** List of all individual plant samples, sorted by metal contents above the limit for aluminum hyperaccumulators (1,000/3,000 mg/kg)

Genus	Species	Organ	*n*	Aluminum content of duplicate measurement in mg/kg			RPD in %	Sample weight in mg
*Landolphia*	*camptoloba*	leaves	6	43,633.3	-	45,065.8	3.23	87.8/101.9
*Landolphia*	*camptoloba*	leaves	6	21,008.2	-	21,533.7	2.47	84.5/78.4
*Anisophyllea*	*quangensis*	leaves	2	20,048.7	-	20,054.2	0.03	405.5/409.5
*Landolphia*	*congolensis*	leaves	1	18,072.1	-	19,683.7	8.54	408.7/401.1
*Commelina*	africana*	leaves	2	LOD	-	15,314.7	-	213.8/185.1
*Landolphia*	*jumellei*	leaves	1	12,890.0	-	15,123.1	15.94	339.9/327.6
*Landolphia*	*lanceolata*	leaves	1	13,875.4	-	14,550.0	4.75	103/104.1
*Euphorbia*	thymifolia*	leaves	1	7,503.5	-	8,934.1	17.41	80.2/78.4
*Anisophyllea*	*quangensis*	leaves	2	7,789.9	-	7,968.4	2.27	401.8/404.4
*Haumaniastrum*	*katangense*	inflorescences	1	4,051.6	-	7,063.3	54.19	11.8/13.1
*Hibiscus*	rhodanthus*	leaves	2	1,962.8	-	6,066.2	102.21	50.3/54.1
*Clerodendrum*	schweinfurthii*	leaves	2	3,939.2	-	5,078.5	25.27	137.2/143.5
*Justicia*	flava*	leaves	1	3,252.6	-	4,819.5	38.82	152.8/135.7
*Commelina*	longifolia*	leaves	2	LOQ	-	4,674.2	-	19.3/11.6
*Landolphia*	*camptoloba*	leaves	6	3,742.9	-	4,138.9	10.05	45.1/62.4
*Spermacoce*	senensis*	leaves	2	2,468.1	-	3,316.4	29.33	-/-
*Solanum*	mauritianum*	leaves	6	2,945.8	-	2,998.6	1.78	146.9/149.4
*Spermacoce*	*dibrachiata*	leaves	2	2,731.9	-	2,977.6	8.61	-/-
*Spermacoce*	*dibrachiata*	leaves	2	2,534.8	-	2,912.4	13.86	86.7/89.8
*Landolphia*	*villosa*	leaves	1	2,223.0	-	2,316.7	4.13	419.3/421.6
*Blepharis*	buchneri*	leaves	2	1,234.9	-	2,277.2	59.35	10.3/13.7
*Clerodendrum*	volubile*	leaves	2	1,986.9	-	2,198.2	10.10	103/101.8
*Spermacoce*	*quadrisulcata*	leaves	1	1,315.9	-	2,176.9	49.30	-/-
*Pityrogramma*	*calomelanos*	leaves	4	1,673.9	-	2,128.5	23.91	-/-
*Hibiscus*	*rhodanthus*	leaves	2	995.6	-	2,104.4	71.54	92.7/91.3
*Spermacoce*	*pusilla*	leaves	1	1,484.2	-	1,860.2	22.49	-/-
*Capparis*	erythrocarpos*	leaves	1	1,219.9	-	1,610.6	27.61	390.3/132.2
*Calotropis*	gigantea*	leaves	1	1,494.5	-	1,561.1	4.36	158.6/118.7
*Ipomoea*	obscura*	leaves	2	1,364.6	-	1,368.1	0.26	118.4/119.1
*Spermacoce*	*senensis*	leaves	2	1,306.5	-	1,363.6	4.28	31.0/37.4
*Euphorbia*	*hirta*	leaves	1	1,302.8	-	1,339.0	2.74	43.4/42.1
*Bulbostylis*	*cardiocarpoides*	leaves	1	348.7	-	1,234.1	111.88	147.7/157.2
*Monosis*	*conferta*	inflorescences	1	846.6	-	1,030.4	19.58	213.8/200.4
*Waltheria*	*indica*	leaves	2	823.1	-	1,011.1	20.50	71.8/68.5
*Evolvulus*	*alsinoides*	leaves, stem^†^	2	623.4	-	1,005.9	46.95	33.8/38.5

List of all individual plant samples with metal content ranges (minimum to maximum measured values and relative percentage difference (RPD)) that at least partially exceed the limit of 1,000 mg/kg for aluminum hyperaccumulators, sorted in descending order by the maximum measured aluminum content in the sample (LOD: values below limit of detection; LOQ: values below limit of quantification), with total number of analyzed species samples, details of the plant organs examined († mixed sample of several organs) and sample weight of duplicates, . Samples with with iron contents above 1,000 mg/kg are marked (*)

The median manganese contents of all measured samples is 244.4 mg/kg, and the mean is 622.4 mg/kg. All samples showed contents below half the hyperaccumulator threshold of 10,000 ppm, except for *Landolphia congolensis*, which had a manganese content of up to 20,895.7 mg/kg.

All of the species identified as aluminum or manganese hyperaccumulators have not been described as hyperaccumulators for that element before (*L. congolensis* has been described as an aluminum hyperaccumulator by Baumgärtel et al. before but with a much lower content < 2,000 mg/kg). Some species, *Calotropis gigantea* (Cu), *Euphorbia thymifolia* (Ni), *Evolvulus alsinoides* (Ni), *Haumaniastrum katagense* (Cu, Co), *Hibiscus rhodanthus* (Cu, Co), *Pityrogramma calomelanos* (As) and *Waltheria indica* (Cu) have been described as hyperaccumulators for other elements.

For all other elements examined, the contents determined in the samples were well below the respective limit values for hyperaccumulators.

### High aluminum and manganese contents – toxicity and tolerance

Aluminum becomes phytotoxic at high contents, particularly in acidic soils (pH < 5), where Al(III) predominates. Toxicity mainly impairs root growth and nutrient uptake by affecting cell division and elongation in root apices. Aluminum interacts with cell-wall components, stiffens cell walls, disrupts Ca(II)-dependent signaling, interferes with cytoskeleton dynamics, reduces DNA replication, and promotes oxidative stress (Kochian et al., 2004; Ofoe et al., 2023; Rout et al., 2001). Additional impacts include reduced chlorophyll content, altered gas exchange, and nuclear DNA damage (Ofoe et al., 2023).

Plants cope with aluminum via exclusion or internal tolerance mechanisms (Kochian et al., 2015). Hyperaccumulators primarily rely on the latter, taking up aluminum, detoxifying it, distributing it, and storing it in tissues. Aluminum stress perception involves protein interactions and receptor-mediated signaling, leading to changes in gene expression (Kochian et al., 2015), which may be governed by either single genes or complex multigene networks (Delhaize et al., 2012).

A central mechanism in both excluders and accumulators is the secretion of organic acids (e.g., oxalate, malate, citrate) as ligands for the complex binding of Al(III) in the rhizosphere (Kochian et al., 2004; Poschenrieder et al., 2015). In accumulators, complexation also facilitates controlled uptake. Aluminum tolerance further involves cell-wall modification, vacuolar sequestration via membrane transporters, and xylem-mediated translocation to shoots, where aluminum is stored in various organelles, including vacuoles, epidermis, cuticle, or chloroplasts (Kochian et al., 2015; Poschenrieder et al., 2015).

Manganese toxicity primarily affects aboveground organs; roots are secondarily damaged and may turn brown (Foy, 2015). Toxicity thresholds vary widely among species (Mukhopadhyay & Sharma, 1991), and symptoms include necrosis, chlorosis, leaf deformation, reduced seed number and viability, and growth inhibition resulting from auxin imbalance and oxidative stress (Kochian et al., 2004; Foy, 2015; Mukhopadhyay & Sharma, 1991; Foy et al., 1978).

Tolerance to manganese may correlate with aluminum tolerance (Foy et al., 1978), but underlying mechanisms are less well established. Current evidence indicates roles for complexation with organic acids such as citrate and oxalate, which reduce ion activity and toxicity (Xu et al., 2008), and for specific transport proteins that mediate Mn(II) sequestration into vacuoles and other compartments (Kochian et al., 2004).

### Metal contents in different plant organs

In addition to the leaves, samples from other organs of the following 10 species were taken from the same herbarium specimen and examined: *Antidesma venosum* (fruits); *Buchnera lippioides* (inflorescences); *Clerodendrum volubile* (fruits); *Dalbergia hostilis* (fruits); *Ficus thonningii* (fruits); *Laportea aestuans* (fruits); *Ochna pulchra* (inflorescences + fruits); *Olax gambecola* (fruits); *Solanum terminale* (inflorescences); *Solanum mauritianum* (inflorescences). With a focus on the elements aluminum, copper, iron, and manganese, content differences between different plant organs were examined in more detail.

The aluminum contents in inflorescences and fruits is, on average, significantly lower than the contents in the leaves. It averages 21.3% (*n* = 7) of the content in the leaves of the respective plant. The fruits contain, on average, 20.9% (*n* = 5), and the inflorescences 22.3% (*n* = 2) of the aluminum content in the leaves.

One particularly striking sample are the flowers of *Haumaniastrum katangense*, with an aluminum content exceeding 7,000 mg/kg. A comparison with leaves is not possible due to a lack of samples but would be very interesting due to the on average higher contents in leaves.

The copper contents in inflorescences and fruits are slightly higher than the content in the leaves. It averages 111.4% (*n* = 7) of the content in the leaves of the respective plant. The fruits contain, on average, 109.5% (*n* = 5), and the inflorescences 144.4% (*n* = 2) of the copper content in the leaves. However, the values vary considerably from plant to plant. For example, the fruits of *Olax gambecola* contain only 35.6% of the copper content in the leaves, while the fruits of *Ficus thonningii* contain 190.3% of the copper content in the leaves.

Inflorescences and fruits contain, on average, less than half of the iron content of the leaves. It averages 43.5% (*n* = 8) of the content in the leaves of the respective plant. The fruits contain, on average, 47.0% (*n* = 6), and the inflorescences 32.8% (*n* = 2) compared to the leaves.

The manganese contents in inflorescences and fruits are, on average, one-third of the content in the leaves. It averages 34.1% (*n* = 11) of the content in the leaves of the respective plant. The fruits contain, on average, 33.4% (*n* = 7), and the inflorescences 29.7% (*n* = 4) of the manganese content in the leaves. The content ratio also varies greatly for manganese; for example, the fruits of *Olax gambecola* contain only 3.5%, while the fruits of *Laportea aestuans* contain 88.7% of the manganese content of the leaves.

In the samples examined, the metal contents of the different samples vary greatly depending on the plant organ or element. However, across all samples and organs, the metal content appears to be lower in fruits and inflorescences than in the leaves. One possible explanation for this is that during plant growth, most metals are transported from the roots to the leaves via the transpiration stream in the xylem, where they are deposited. Later redistribution to flowers and fruits occurs via the phloem, against the transpiration stream. The amount of metal translocated depends heavily on their mobility in the phloem. Therefore, metals often accumulate more strongly in older leaves than in younger leaves or short-lived organs such as flowers and fruits (Page & Feller, 2015).

### Site influence on the metal content for different individuals of a species

The collection site of the specimens plays a crucial role in the search for hyperaccumulators. Although certain species are capable of accumulating specific metals in amounts exceeding hyperaccumulator thresholds, this ability is highly dependent on the type of the metal, its chemical speciation, and its bioavailability to the plant. This is well illustrated by aluminum hyperaccumulating species: in cases where multiple samples of a single species were analyzed, contents exceeding the threshold were detected in 17 out of 31 samples. This corresponds to a frequency of 54.8% above the hyperaccumulation threshold. These findings suggest that a considerable proportion of species not yet classified as hyperaccumulators, due to only one or few samples being analyzed, may potentially possess the capacity for hyperaccumulation. A vivid example of this is the species *Landolphia camptoloba* where contents range from below LOQ up to 43,633.3—45,065.8 mg/kg in another sample, i.e., several hundred times higher. This trend can also be seen with other metals over various species (Table 3).

**Table 3:** Variability of metal contents in plant samples from different locations

Element	Number of samples	Number of species	SD in mg/kg	RSD
Al	103	38	907.7	61.8%
Cu	78	28	2.9	22.3%
Fe	117	43	162.1	48.6%
Mn	130	47	310.5	48.3%
Ni	45	19	1.7	36.1%

Number of samples and species analyzed for the elements aluminum, copper, iron, manganese, and nickel, as well as the mean standard deviation (SD) and mean relative standard deviation (RSD) of element contents between samples of the same species from different locations. Cadmium, cobalt, chromium, lead, and zinc are not considered due to the small number of samples with quantifiable contents

Certain discrepancies between herbarium analyses and records in the Global Hyperaccumulator Database highlight the strong influence of collection site as well. Several species listed there as cobalt or copper hyperaccumulators (e.g., *Acalypha cupricola*, *Anisopappus chinensis*, *Buchnera henriquesii*) did not show elevated metal contents in the examined herbarium specimens. Likewise, species in this study identified as aluminum hyperaccumulators (e.g. *Calotropis gigantea*, *Euphorbia thymifolia*, *Evolvulus alsinoides*, *Haumaniastrum katangense*, *Hibiscus rhodanthus*) were not reported as aluminum hyperaccumulators but as cobalt, copper, or nickel hyperaccumulators instead.

**Fig. 3:**
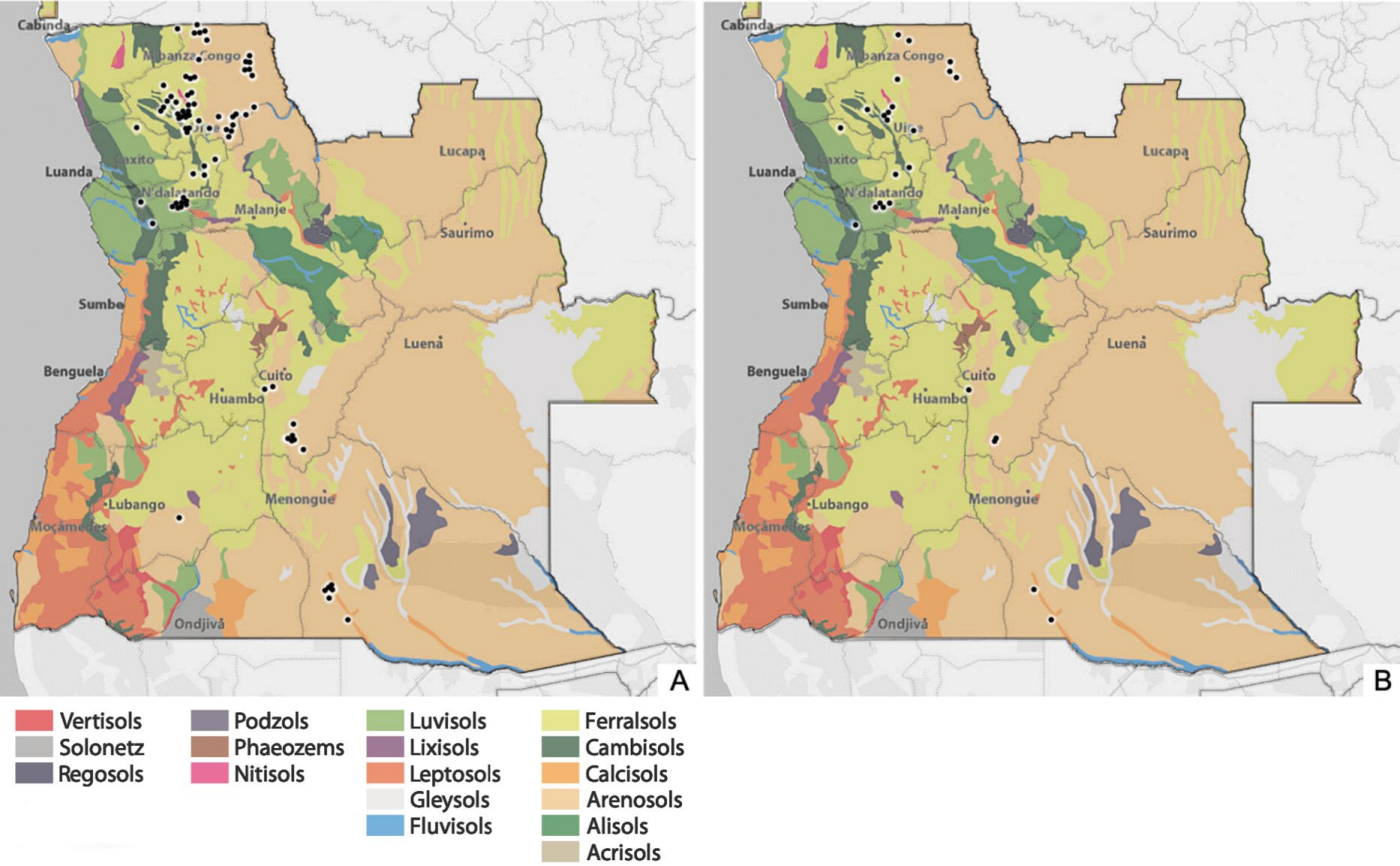
Map of the herbarium specimens showing the soil types of Angola. Map of the soil types in Angola (Huntley, 2019), showing the locations of all the herbarium specimens examined (**A**), and showing the locations of the specimens identified as hyperaccumulators (**B**) (Map excerpt underlying the soil map: Data and rendering from Google Maps. Map data ©2025 Google, GeoBasis-DE/BKG (©2025))

Herbarium specimens collected from soils classified as Arenosols, Cambisols, Ferralsols, Nitisols, and Phaeozems were analyzed, and hyperaccumulator species were identified on all these soil types. The majority of aluminum hyperaccumulators were found on Arenosols, Ferralsols, and Phaeozems, which were also the soil types on which most of the specimens were collected (Fig. 3). These soils are highly weathered and leached with low clay contents, often showing little concentrations of the targeted metals, mainly dominated by stable aluminum and iron compounds (Joint Research Centre of the European Commission, 2013). This correlates well with the findings mainly dominated by aluminum hyperaccumulators. Further analyzes of the Soil and metal contents in it were not done due to the unavailability of soil samples from the collection sites.

### Potential use for phytomining

The economic feasibility of metal extraction using hyperaccumulator plants depends mainly on global market prices of the metals, as well as soil metal contents, accumulated contents in plant tissues, annual biomass production, and the costs of cultivation, harvesting, and extraction (Sheoran et al., 2009). In 2022, average prices were 2,706 USD per ton of aluminum and 3,167.90 USD per ton of manganese, classifying them as relatively low-value metals (Statista, 2024).

Based on the highest measured leaf contents, very large biomass amounts would be required to obtain metals worth 1,000 USD. For *Landolphia camptoloba*, with 45,000 mg/kg aluminum in leaf dry mass, about 369.55 kg of aluminum would correspond to 1,000 USD, requiring approximately 8,212 kg of dry leaf biomass. For *Landolphia congolensis*, with 20,000 mg/kg manganese in leaf dry mass, about 315.67 kg of manganese would correspond to 1,000 USD, requiring roughly 15,784 kg of dry leaf biomass.

Other plant parts, including latex present in many *Landolphia* species, may also be of interest, and contents can vary strongly between organs, plant age, and growth conditions (NRC, 2008). Thus, these calculations represent only rough estimates. Harvesting entire plants, only leaves, or latex could be considered; repeated harvesting might be possible without replanting.

Estimating required cultivation area remains difficult because biomass yields per hectare are unknown and feasibility of monoculture has not been studied. *L. camptoloba*, a climbing shrub, would likely require support structures to achieve high biomass production. Profitability must also include costs of cultivation, maintenance, harvesting, extraction, and purification. Possible co-benefits include energy recovery from combustion during extraction and the sale of fruits of *Anisophyllea* and *Landolphia* species (NRC, 2008). Although elevated aluminum contents have been detected in fruits of *L. congolensis*, no health risk has been reported (Baumgärtel et al., 2023). If latex is unsuitable for metal extraction, it could potentially be used for rubber production, as in *L. owariensis*. Aside from its economical use, plants that are able to accumulate and tolerate such high amounts of metal could serve as interesting model organisms to study metal-plant interactions and offer approaches for crop plants on soils which would otherwise not be suitable for cultivation.

### Method evaluation

Herbarium material offers key advantages over fresh field samples, including access to a wide range of already-identified species in one location and the possibility of laboratory-based analysis. Multiple individuals of the same species from different sites are often available. However, due to the time and resources required for sample preparation and analysis, preselection of specimens is usually necessary. This can be based on taxonomic relationships with known hyperaccumulators or on identifying metal-rich soils and special habitats such as mining areas.

The main limitation of herbarium specimens is the frequent lack of detailed site information beyond basic coordinates, which restricts environmentally guided selection. Another issue is that specimens are typically chosen to be morphologically representative, not for optimal chemical analysis; for hyperaccumulator studies it is important to know from which plant part and at what age samples were taken, as these factors strongly influence metal content (Losfeld et al., 2015).

While ICP-OES enables rapid, precise multi-element quantification, its sample preparation is time- and resource-intensive. Simple qualitative prescreening tests exist for some elements such as Al, Cu, Ni, and Zn (Purwadi et al., 2021). Portable X-ray fluorescence (XRF) allows fast, non-destructive screening of herbarium specimens, though cost and safety requirements currently limit its use (Purwadi et al., 2021). An optimal strategy would combine XRF for large-scale screening with ICP-OES for confirmation and precise quantification.

## Conclusion

This study demonstrates that herbarium collections provide an effective resource for large scale screening of potential metal hyperaccumulator plants. By analyzing 234 specimens representing 132 species, we identified several previously unreported aluminum hyperaccumulators and one manganese hyperaccumulator, with particularly high contents in species of the genus *Landolphia* and notable intraspecific variability linked to collection site. According to the Global Hyperaccumulator Database Blepharis buchneri*, *Bulbostylis cardiocarpoides*, Calotropis gigantea* (Cu), Capparis erythrocarpos*, Clerodendrum schweinfurthii*, Clerodendrum volubile*, Commelina africana*, Commelina longifoia*, *Euphorbia hirta*, Euphorbia thymifolia* (Ni), *Evolvulus alsinoides* (Ni), *Haumaniastrum katangense* (Cu, Co), *Hibiscus rhodanthus* (Cu, Co), Ipomoea obscura*, Justicia flava*, *Landolphia camptoloba*, *Landolphiacongolensis*, *Landolphia jumellei*, *Landolphia lanceolata*, *Landolphia villosa*, *Monosis conferta*, *Pityrogramma calomelanos* (As), Solanum mauritianum*, *Spermacoce dibrachiata*, *Spermacoce pusilla*, *Spermacoce quadrisulcata*, *Spermacoce senensis* and *Waltheria indica* (Cu) are newly described aluminum hyperaccumulators (species marked with (*) should be treated as potential aluminum hyperaccumulators as they also showed iron contents > 1,000 mg/kg; species with elements in brackets were already listed as hyperaccumulators for that specific element; *L. congolensis* and *L. lanceolata* has been described as an aluminum hyperaccumulator by Baumgärtel et al. before but with a much lower content < 2,000 mg/kg). The Species *Landolphia congolensis* is a newly described manganese hyperaccumulator. Aluminum accumulation clearly dominated over other elements, while contents of cadmium, cobalt, chromium, lead, and zinc generally remained below LOD or LOQ. Manganese hyperaccumulation was rare and was confirmed only for *Landolphia congolensis*. Across species, metal contents were typically higher in leaves than in other organs, underscoring organ-specific partitioning of metals within plants.

The main advances provided by this study are twofold. First, it expands the known geographic and taxonomic diversity of hyperaccumulator species in sub-Saharan Africa, providing the first systematic herbarium-based survey for Angola. Second, it establishes that hyperaccumulation capacity is strongly site-dependent, highlighting the need to consider edaphic context and intraspecific variation when classifying hyperaccumulators. These findings deliver a robust basis for future field validation, ecological and physiological studies of metal tolerance, as well as the assessment of potential applications in phytoremediation, phytomining, and environmental monitoring in metal-rich or degraded tropical landscapes.

## Supplementary Information

Below is the link to the electronic supplementary material.

ESM 1 Supplementary Material 1 (DOCX 139 KB)

## Data Availability

For the identification of potential hyper accumulator species we used the Global Hyperaccumulator Database of the Centre for Mined Land Rehabilitation (University of Queensland, Australia). This was formerly a public online database,, however was not accessible at the beginning of the work on this project. We received the database through personal communication with the Institution. Data on the collection location of the herbarium specimens were available on the specimens itself. The specimens can be viewed via the online database JACQ, the specimen numbers can be obtained through personal communication with the authors of this work.

## Declarations

All authors have read, understood, and have complied as applicable with the statement on “Ethical responsibilities of Authors” as found in the Instructions for Authors.
