# Supplementary material for: Screening for potential metal hyperaccumulator plants in Angola: a herbarium-based approach
Source: Environ Monit Assess. 2026 Aug 1;198(8):899. doi: 10.1007/s10661-026-15744-w (PMC13428701; doi:10.1007/s10661-026-15744-w)
Supplement: Supplementary file 1 — Supplementary Material 1 (DOCX 139 KB) [file 10661_2026_15744_MOESM1_ESM.docx]

**Supplement 1**

**Table S1: Element contents of the species studied**

List of all species examined, sorted by family, genus, species with information on analyzed plant organ (le = leaves; fr = fruits; in = inflorescences; tu = tuber, st = stem axis, mixed samples if more than one organ is specified) and growth form ((H) = herb; (S) shrub; (sS) subshrub; (T) tree; (L) liana; (a) annual; (ba) biennial; (p) perennial). The lowest and highest measured values (in mg/kg based on plant dry mass) are given for the following elements (measurement wavelength): aluminum (396.152 nm), cadmium (214.439 nm), cobalt (238.892 nm), chromium (205.560 nm), copper (324.754 nm), iron (259.940 nm), manganese (259.372 nm), nickel (231.604 nm), lead (182.143 nm) and zinc (213.857 nm). Measured values for species marked with (*) are composed of samples from different locations, values marked with (‡) were determined using the standard addition method. LOD: values are below the limit of detection, LOQ: values are below the limit of quantification (above LOD). Species in bold print, with the underlined contents, are above the limit for hyperaccumulators of the respective element. Species marked with (#) included samples with white precipitate after digestion.

|  |  |  |  |  | **Al** | | **Cd** | | **Co** | | **Cr** | | **Cu** | | **Fe** | | **Mn** | | **Ni** | | **Pb** | | **Zn** | |
| --- | --- | --- | --- | --- | --- | --- | --- | --- | --- | --- | --- | --- | --- | --- | --- | --- | --- | --- | --- | --- | --- | --- | --- | --- |
| **family** | **genus** | **species** | **organ** | **growth** | min. | max. | min. | max. | min. | max. | min. | max. | min. | max. | min. | max. | min. | max. | min. | max. | min. | max. | min. | max. |
| Acanthaceae | *Blepharis* | *buchneri*^#^* | le | S | 36.1 | 2,277.2^‡^ |  | LOD |  | LOD |  | LOD | LOQ | LOQ | 72.1 | 1,456.3 | 264.4 | 1,213.6 | LOQ | LOQ |  | LOD | LOQ | LOQ |
| Acanthaceae | ***Justicia*** | ***flava****^#^* | le | pH | 3,252.6 | 4,819.5 |  | LOD |  | LOD | 6.5 | 14.7 | LOQ | LOQ | 2,745.4 | 4,469.4 | 88.4 | 117.9 | LOQ | LOQ | LOD | LOQ | LOQ | LOQ |
| Adianthaceae | *Pteris* | spec.^#^ | le | - | 137.2 | 142.3 |  | LOD |  | LOD |  | LOD | LOQ | LOQ | 174.6 | 186.7 | 311.7 | 355.7 | LOQ | LOQ |  | LOD | LOQ | LOQ |
| Anisophylleaceae | ***Anisophyllea*** | ***quangensis*****^#^* | le | S | 7,789.9^‡^ | 20,054.2^‡^ |  | LOD |  | LOD | 1.2 | 2.5 | LOQ | LOQ | 65.9 | 77.7 | 204.0 | 794.1 | LOQ | LOQ |  | LOD |  | LOD |
| Apocynaceae | ***Calotropis*** | ***gigantea****^#^* | le | S/T | 1,494.5^‡^ | 1,561.1^‡^ |  | LOD |  | LOD | 3.2 | 4.2 | 6.3 | 8.4 | 1,406.1 | 1,676.5 | 119.8 | 155.9 | LOD | 4.2 | LOD | LOQ |  | LOD |
| Apocynaceae | *Clitandra* | *cymulosa^#^* | le | L | 609.0 | 611.2 |  | LOD |  | LOD |  | LOD | 13.5 | 14.3 | 80.0 | 83.5 | 532.6 | 619.8 | 4.9 | 6.0 |  | LOD | 27.0 | 32.2 |
| Apocynaceae | *Landolphia* | *buchananii^#^* | le | S/L | 69.9 | 70.0 |  | LOD |  | LOD |  | LOD | 19.6 | 20.0 | 38.7 | 40.5 | 525.7 | 575.6 | 2.5 | 2.5 |  | LOD | LOQ | LOQ |
| Apocynaceae | ***Landolphia*** | ***camptoloba*****^#^* | le | S | LOQ | 45,065.8^‡^ | LOD | 1.2 |  | LOD | LOD | 2.4 | LOQ | 10.4 | LOQ | 96.3 | 144.1 | 2,562.4 | LOD | 2.4 | LOD | 3.6 | LOD | LOQ |
| Apocynaceae | ***Landolphia*** | ***congolensis****^#^* | le | L | 18,072.1^‡^ | 19,683.7^‡^ | 2.4 | 2.5 | 4.9 | 5.0 | 1.2 | 1.2 | 11.0 | 11.2 | 144.4 | 145.8 | 20,876.3^‡^ | 20,895.7^‡^ | 7.3 | 7.5 | 19.9 | 20.8 | 121.1 | 130.9 |
| Apocynaceae | ***Landolphia*** | ***jumellei****^#^* | le | S/L | 12,890.0^‡^ | 15,123.1^‡^ |  | LOD |  | LOD | LOD | LOQ | 10.7 | 11.8 | 64.7 | 74.8 | 738.5 | 815.0 | 2.9 | 3.1 |  | LOD | LOQ | 29.4 |
| Apocynaceae | ***Landolphia*** | ***lanceolata*** | le | sS | 13,875.4^‡^ | 14,550.0^‡^ |  | LOD |  | LOD |  | LOD | 9.6 | 9.7 | 52.8 | 53.4 | 341.0 | 349.5 | 4.8 | 4.9 | LOD | LOQ |  | LOD |
| Apocynaceae | *Landolphia* | *owariensis*^#^* | le | L | 372.8 | 640.9 |  | LOD |  | LOD | 1.2 | 1.2 | 7.2 | 13.5 | 49.2 | 198.4 | 1,096.2 | 1,380.5 | 4.9 | 12.1 |  | LOD | LOQ | 27.1 |
| Apocynaceae | *Landolphia* | *robustior^#^* | le | L | 270.3 | 303.0 |  | LOD |  | LOD | LOD | LOQ | LOQ | LOQ | 77.4 | 78.0 | 1,999.3 | 2,023.8 | 1.2 | 1.3 |  | LOD | LOQ | 28.3 |
| Apocynaceae | ***Landolphia*** | ***villosa*** | le | L | 2,223.0^‡^ | 2,316.7^‡^ |  | LOD |  | LOD |  | LOD | 7.1 | 7.2 | 50.1 | 55.7 | 441.2 | 476.8 | 1.2 | 1.2 |  | LOD | LOQ | LOQ |
| Asparagaceae | *Ledebouria* | spec. | le | - |  | LOD |  | LOD |  | LOD |  | LOD |  | LOD |  | LOD | 43.5 | 50.0 |  | LOD |  | LOD |  | LOD |
| Asparagaceae | *Ledebouria* | spec. | tu | - | LOD | 153.5 |  | LOD |  | LOD |  | LOD | LOD | LOQ | LOD | LOQ | 18.7 | 65.8 |  | LOD |  | LOD |  | LOD |
| Asteraceae | *Anisopappus* | *buchwaldii^#^* | le | pH | 100.0 | 125.1 |  | LOD |  | LOD |  | LOD | LOQ | LOQ | 105.0 | 125.1 | 320.0 | 360.9 |  | LOD |  | LOD |  | LOD |
| Asteraceae | *Anisopappus* | *chinensis^#^* | le | pH | 98.9 | 109.3 |  | LOD |  | LOD |  | LOD | LOQ | LOQ | 74.2 | 76.5 | 672.1 | 766.6 |  | LOD |  | LOD | LOD | LOQ |
| Asteraceae | *Berkheya* | *angolensis*^#^* | le | pH | 63.0 | 698.6 |  | LOD |  | LOD | LOD | 2.3 | 6.9 | 15.9 | 66.7 | 558.9 | 130.8 | 1,434.4 | LOD | 5.8 |  | LOD | LOD | 45.9 |
| Asteraceae | *Bidens* | *buchneri^#^* | le | H/S | 117.2 | 135.1 |  | LOD |  | LOD |  | LOD | 8.4 | 8.8 | 94.9 | 108.7 | 229.1 | 234.4 |  | LOD |  | LOD | LOQ | LOQ |
| Asteraceae | *Chromolaena* | *odorata** | le, in | S | 86.3 | 601.4 |  | LOD |  | LOD | 1.7 | 3.2 | 13.9 | 22.6 | 74.8 | 404.8 | 37.4 | 198.5 | 2.9 | 6.9 |  | LOD | LOQ | LOQ |
| Asteraceae | *Gymnanthemum* | *amygdalinum** | le | S/T | 31.9 | 106.8 |  | LOD |  | LOD | 1.2 | 2.4 | 10.8 | 18.4 | 63.5 | 119.5 | 44.3 | 89.7 | LOQ | LOQ |  | LOD | LOQ | 33.6 |
| Asteraceae | *Gymnanthemum* | *amygdalinum* | in | S/T | 494.9 | 713.8 |  | LOD |  | LOD | 1.2 | 1.5 | 23.2 | 23.7 | 434.0 | 524.4 | 112.1 | 114.7 | LOQ | LOQ |  | LOD | 34.9 | 46.4 |
| Asteraceae | ***Monosis*** | ***conferta*** | in | T | 846.6 | 1,030.4 |  | LOD |  | LOD | 2.3 | 2.5 | 9.4 | 10.0 | 930.8 | 978.0 | 205.8 | 207.1 | LOQ | LOQ |  | LOD |  | LOD |
| Asteraceae | *Nidorella* | *attenuata* | le | aH | 365.5 | 434.5 |  | LOD |  | LOD | 1.2 | 1.2 | 9.8 | 9.8 | 241.2 | 278.2 | 296.6 | 302.8 | LOQ | LOQ |  | LOD | 41.8 | 45.5 |
| Asteraceae | *Senecio* | *ornatus* | le | - | 99.4 | 109.5 |  | LOD |  | LOD | 1.5 | 1.6 | 4.5 | 4.8 | 53.4 | 58.7 | 215.2 | 279.4 | 3.0 | 3.2 |  | LOD |  | LOD |
| Cannabaceae | *Trema* | *orientale** | le | S/T | 94.0 | 303.1 |  | LOD |  | LOD | 1.4 | 2.6 | 4.1 | 5.2 | 96.5 | 233.1 | 145.1 | 183.9 | LOQ | LOQ |  | LOD | LOD | LOQ |
| Capparaceae | ***Capparis*** | ***erythrocarpos****^#^* | le | S | 1,219.9^‡^ | 1,610.5^‡^ |  | LOD |  | LOD | 3.6 | 6.4 | 4.8 | 5.1 | 1,498.2 | 2,587.8 | 134.2 | 161.4 | 2.4 | 3.8 |  | LOD | 27.8 | 33.3 |
| Clusiaceae | *Clusia* | *huillensis* | le | - | LOQ | LOQ |  | LOD |  | LOD |  | LOD | LOQ | LOQ | 68.8 | 77.3 | 463.6 | 663.1 |  | LOD |  | LOD | LOQ | LOQ |
| Clusiaceae | *Garcinia* | *huillensis* | le | S/T | 86.6 | 98.3 |  | LOD |  | LOD |  | LOD | 10.8 | 11.3 | 72.2 | 79.4 | 735.9 | 938.0 | 3.6 | 3.8 |  | LOD |  | LOD |
| Clusiaceae | *Garcinia* | *kola* | le | T | 44.7 | 53.3 |  | LOD |  | LOD |  | LOD | LOQ | LOQ | 44.7 | 49.9 | 506.0 | 577.7 | 3.3 | 3.4 |  | LOD |  | LOD |
| Commelinaceae | ***Commelina*** | ***africana*****^#^* | le | pH | LOD^‡^ | 15,314.7^‡^ |  | LOD |  | LOD | LOD | 10.8 | LOD | 13.5 | LOQ | 8,833.9^‡^ | 552.3 | 761.8 | LOD | 8.1 | LOD | LOQ | LOD | LOQ |
| Commelinaceae | *Commelina* | *capitata*^#^* | le | sS | 78.0 | 215.7 |  | LOD |  | LOD |  | LOD | LOQ | 12.6 | 104.0 | 215.7 | 213.4 | 2,241.8 | 3.4 | 6.5 |  | LOD |  | LOD |
| Commelinaceae | ***Commelina*** | ***longifolia^#^*** | le | sS | LOQ | 4,674.2^‡^ |  | LOD |  | LOD |  | LOD |  | LOD | LOQ | 5,170.3^‡^ | 880.8 | 2,241.4 |  | LOD |  | LOD |  | LOD |
| Commelinaceae | *Commelina* | *longifolia^#^* | le, st | sS | 216.1 | 244.3 |  | LOD |  | LOD |  | LOD | LOQ | LOQ | 112.2 | 138.9 | 16.6 | 19.2 |  | LOD |  | LOD |  | LOD |
| Commelinaceae | *Cyanotis* | *pilosa^#^* | le | pH | 280.7 | 371.9 |  | LOD |  | LOD |  | LOD |  | LOD | 207.9 | 216.9 | 1,746.4 | 2,345.0 |  | LOD |  | LOD | LOD | LOQ |
| Convolvulaceae | ***Evolvulus*** | ***alsinoides****^#^* | le, st | a/pH | 623.4 | 1,005.9 |  | LOD |  | LOD |  | LOD |  | LOD | 142.9 | 236.7 | 675.3 | 680.5 |  | LOD |  | LOD |  | LOD |
| Convolvulaceae | *Evolvulus* | *alsinoides^#^* | bl, in, st | a/pH | 82.1 | 114.7 |  | LOD |  | LOD |  | LOD | LOD | LOQ | 82.1 | 103.2 | 103.2 | 127.7 |  | LOD |  | LOD |  | LOD |
| Convolvulaceae | *Iponema* | *batatas^#^* | le | H | 341.2 | 397.8 |  | LOD |  | LOD |  | LOD | 13.9 | 14.3 | 215.9 | 268.8 | 104.5 | 136.2 |  | LOD |  | LOD |  | LOD |
| Convolvulaceae | ***Ipomoea*** | ***obscura*****^#^* | le | a/pH | 1,364.6^‡^ | 1,368.1^‡^ |  | LOD |  | LOD | 4.2 | 8.4 | 8.4 | 8.4 | 1,083.1 | 1,237.3 | 570.9 | 595.4 | 4.2 | 4.2 |  | LOD |  | LOD |
| Convolvulaceae | *Ipomoea* | *obscura^#^* | le, fr, st | a/pH | LOQ | LOQ |  | LOD |  | LOD |  | LOD | LOQ | LOQ | LOQ | LOQ | 156.3 | 216.7 |  | LOD |  | LOD |  | LOD |
| Convolvulaceae | *Ipomoea* | *prismatosyphon* | le | (s)S | 306.0 | 316.0 |  | LOD |  | LOD |  | LOD | 5.1 | 5.1 | 209.0 | 229.5 | 109.6 | 127.5 |  | LOD |  | LOD |  | LOQ |
| Convolvulaceae | *Merremia* | *pterygocaulos* | le | (s)S | 229.4 | 244.4 |  | LOD |  | LOD |  | LOD | LOQ | LOQ | 200.0 | 213.0 | 119.0 | 129.4 |  | LOD |  | LOD |  | LOD |
| Cyperaceae | ***Bulbostylis*** | ***cardiocarpoides*** | le | pH | 348.7 | 1,234.1 |  | LOD |  | LOD |  | LOD | LOQ | LOQ | 247.1 | 820.6 | 419.8 | 451.7 | 3.2 | 3.4 |  | LOD |  | LOD |
| Cyperaceae | *Bulbostylis* | *filamentosa* | entire pl. | pH | 67.9 | 519.3 |  | LOD |  | LOD | LOD | 1.2 | 2.4 | 2.4 | 54.6 | 372.5 | 108.0 | 126.6 | 1.2 | 1.2 |  | LOD |  | LOD |
| Dichapetalaceae | *Dichapetalum* | *heudelotii* | le | S/T | 330.8 | 480.9 |  | LOD |  | LOD |  | LOD | LOQ | LOQ | 261.5 | 320.6 | 93.5 | 101.4 |  | LOD |  | LOD |  | LOD |
| Ebenaceae | *Diospyros* | *heterotricha** | le | T | 56.3 | 460.8 |  | LOD |  | LOD |  | LOD | 5.0 | 7.6 | 50.1 | 222.8 | 238.6 | 436.1 | 2.0 | 5.0 |  | LOD |  | LOD |
| Ebenaceae | *Diospyros* | *pseudomespilus** | le | T | LOQ | 772.6 |  | LOD |  | LOD |  | LOD | LOQ | LOQ | 381.7 | 444.0 | 400.7 | 6,336.9^‡^ | LOD | 35.5 |  | LOD | LOD | LOQ |
| Euphorbiaceae | *Acalypha* | *ciliata* | le | aH | LOQ | 103.2 |  | LOD |  | LOD |  | LOD | LOQ | LOQ | 70.6 | 127.0 | 127.0 | 141.3 |  | LOD |  | LOD |  | LOD |
| Euphorbiaceae | *Acalypha* | *cupricola^#^* | le | S | 190.9 | 191.5 |  | LOD |  | LOD |  | LOD | LOQ | LOQ | 78.0 | 80.9 | 173.8 | 184.5 |  | LOD |  | LOD |  | LOD |
| Euphorbiaceae | *Acalypha* | *ornata** | le | S | 266.0 | 702.9 |  | LOD |  | LOD |  | LOD | 9.5 | 13.8 | 291.3 | 702.9 | 50.7 | 96.8 |  | LOD |  | LOQ | LOQ | LOQ |
| Euphorbiaceae | *Acalypha* | *paniculata* | le | S | LOQ | LOQ |  | LOD |  | LOD |  | LOD | LOQ | LOQ | 88.7 | 98.3 | 87.3 | 88.7 |  | LOD |  | LOD |  | LOD |
| Euphorbiaceae | *Acalypha* | *welwitschiana^#^* | le | sS | 153.5 | 162.2 |  | LOD |  | LOD |  | LOD | 6.0 | 6.1 | 123.6 | 127.7 | 213.3 | 221.0 |  | LOD |  | LOD | LOQ | LOQ |
| Euphorbiaceae | *Croton* | *gratissimus*^#^* | le | S/T | 46.7 | 85.1 |  | LOD |  | LOD |  | LOD | 8.5 | 9.0 | 55.2 | 125.4 | 632.4 | 902.2 | LOD | 4.5 |  | LOD |  | LOD |
| Euphorbiaceae | *Croton* | *mubango*^#^* | le | T | LOQ | 464.1 |  | LOD |  | LOD |  | LOD | 5.7 | 6.4 | 57.9 | 435.8 | 116.0 | 182.6 | LOD | 3.2 |  | LOD | LOD | LOQ |
| Euphorbiaceae | *Croton* | *sylvaticus^#^* | le, in | T | 54.9 | 61.9 |  | LOD |  | LOD |  | LOD | 8.7 | 9.9 | 57.8 | 61.9 | 497.4 | 564.9 | LOD | 2.5 |  | LOD | LOQ | LOQ |
| Euphorbiaceae | ***Euphorbia*** | ***hirta****^#^* | le | aH | 1,302.8^‡^ | 1,339.0^‡^ |  | LOD |  | LOD |  | LOD | LOQ | LOQ | 668.2 | 688.8 | 23.0 | 23.8 |  | LOD |  | LOD | LOD | 380.0 |
| Euphorbiaceae | *Euphorbia* | *pulcherrima^#^* | le | S | LOQ | LOQ |  | LOD |  | LOD |  | LOD | 9.1 | 10.0 | 72.5 | 80.1 | 31.7 | 35.0 |  | LOD |  | LOD | LOQ | LOQ |
| Euphorbiaceae | ***Euphorbia*** | ***thymifolia****^#^* | le | aH | 7,503.5^‡^ | 8,934.1^‡^ |  | LOD |  | LOD | 6.2 | 6.4 | LOQ | LOQ | 5,367.5^‡^ | 6,506.6^‡^ | 49.9 | 51.0 |  | LOD | LOD | LOQ | LOD | LOQ |
| Euphorbiaceae | *Macaranga* | *angolensis*^#^* | le | S/T | 15.5 | 800.2 |  | LOD |  | LOD | LOD | 1.3 | 7.6 | 13.9 | 40.5 | 602.5 | 99.6 | 664.7 | LOD | 7.8 |  | LOD | LOD | LOQ |
| Euphorbiaceae | *Macaranga* | *monandra*^#^* | le | T | 54.1 | 589.5 |  | LOD |  | LOD |  | LOD | 4.4 | 11.3 | 73.6 | 387.2 | 259.2 | 1,219.1 | LOD | 2.6 | LOD | LOQ | LOD | LOQ |
| Euphorbiaceae | *Macaranga* | *schweinfurthii^#^* | le | T | 33.5 | 40.9 |  | LOD |  | LOD |  | LOD | 5.0 | 5.1 | 70.6 | 74.2 | 133.8 | 140.7 | LOD | 1.3 |  | LOD |  | LOD |
| Euphorbiaceae | *Macaranga* | *spinosa* | le | T | 52.5 | 57.5 |  | LOD |  | LOD |  | LOD | 5.5 | 6.1 | 71.9 | 75.7 | 44.2 | 54.5 |  | LOD |  | LOD | LOD | LOQ |
| Fabaceae | *Crotalaria* | *lundensis^#^* | le | pH | 129.9 | 133.7 |  | LOD |  | LOD |  | LOD | LOQ | LOQ | 102.0 | 104.0 | 163.4 | 185.5 |  | LOD |  | LOD |  | LOD |
| Fabaceae | *Crotalaria* | *ochroleuca* | le | a/pH/sS | LOQ | LOQ |  | LOD |  | LOD |  | LOD | 15.1 | 15.6 | 78.0 | 85.6 | 124.9 | 130.9 |  | LOD |  | LOD |  | LOD |
| Fabaceae | *Dalbergia* | *carringtoniana* | le | T | 30.6 | 31.5 |  | LOD |  | LOD |  | LOD | 4.2 | 4.7 | 56.4 | 56.7 | 101.1 | 104.9 | LOD | 2.1 |  | LOD | LOQ | LOQ |
| Fabaceae | *Dalbergia* | *hostilis*^#^* | le | S/L | 104.6 | 564.9 |  | LOD |  | LOD |  | LOD | 19.2 | 27.9 | 133.2 | 592.8 | 100.8 | 325.1 | 1.9 | 6.2 | LOD | LOQ | LOQ | 108.6 |
| Fabaceae | *Dalbergia* | *nitidula** | le | S/T | LOQ | 81.3 |  | LOD |  | LOD |  | LOD | LOQ | 5.4 | 25.1 | 70.7 | 285.6 | 724.4 | LOD | 1.8 |  | LOD | LOQ | 39.7 |
| Fabaceae | *Dalbergia* | *oligophylla* | le | S | 76.3 | 82.2 |  | LOD |  | LOD |  | LOD | 5.1 | 5.1 | 71.2 | 75.4 | 381.4 | 479.6 |  | LOD |  | LOD | LOQ | LOQ |
| Fabaceae | *Eriosema* | *shirense* | le | pH | LOD | LOQ |  | LOD |  | LOD |  | LOD |  | LOD | LOQ | LOQ | 35.1 | 78.4 |  | LOD |  | LOD |  | LOD |
| Fabaceae | *Sesbania* | *macrantha* | le | H/S/T | 78.2 | 100.1 |  | LOD |  | LOD |  | LOD | 7.4 | 10.7 | 141.5 | 168.0 | 85.6 | 100.1 |  | LOD |  | LOD | LOQ | LOQ |
| Fabaceae | *Sesbania* | *sesban** | le | S/T | LOD | LOQ |  | LOD |  | LOD |  | LOD | LOQ | 9.0 | LOQ | 80.9 | 94.8 | 293.4 | LOD | 11.1 |  | LOD | LOD | LOQ |
| Fabaceae | *Tephrosia* | *nana** | le | a/ph | 54.3 | 638.3 |  | LOD |  | LOD |  | LOD | 4.4 | 9.3 | 74.7 | 450.3 | 83.4 | 531.6 | LOD | 2.3 |  | LOD | LOD | LOQ |
| Fabaceae | *Tephrosia* | *vogelii** | le | pH/S | 80.3 | 418.6 |  | LOD |  | LOD |  | LOD | LOQ | 8.5 | 96.7 | 335.4 | 37.0 | 1,235.8 |  | LOD |  | LOD | LOD | LOQ |
| Lamiaceae | *Clerodendrum* | *formicarum*^#^* | le | S/T | 151.2 | 668.0 |  | LOD |  | LOD | LOD | 3.4 | LOQ | 10.6 | 84.5 | 413.8 | 186.0 | 859.4 | LOD | 7.0 |  | LOD | LOD | LOQ |
| Lamiaceae | *Clerodendrum* | *globuliflorum* | le | S | 80.1 | 92.9 |  | LOD |  | LOD |  | LOD | 7.3 | 7.3 | 78.3 | 85.6 | 919.9 | 941.7 | 1.8 | 1.8 |  | LOD |  | LOD |
| Lamiaceae | ***Clerodendrum*** | ***schweinfurthii**** | le | S/L | 251.5 | 5,078.5^‡^ |  | LOD |  | LOD | LOD | 4.7 | 8.4 | 32.8 | 223.0 | 3,083.8 | 185.9 | 594.0 | 2.1 | 7.3 | LOD | LOQ | LOQ | LOQ |
| Lamiaceae | *Clerodendrum* | *silvanum* | le | S/L | 90.9 | 93.2 |  | LOD |  | LOD | LOD | LOD | 11.7 | 13.0 | 98.7 | 99.1 | 1,231.2 | 1,235.4 | 2.9 | 5.2 |  | LOD | LOD | LOQ |
| Lamiaceae | ***Clerodendrum*** | ***volubile*** | le | S/L | 1,986.9^‡^ | 2,198.2^‡^ |  | LOD |  | LOD | 4.9 | 4.9 | LOQ | LOQ | 1,689.3 | 1,969.5 | 1,339.8 | 1,360.5 | 4.9 | 4.9 |  | LOD |  | LOD |
| Lamiaceae | *Clerodendrum* | *volubile* | fr | S/L | 154.3 | 199.2 |  | LOD |  | LOD |  | LOD | 10.3 | 10.9 | 162.1 | 195.3 | 213.4 | 229.1 | 7.8 | 7.8 |  | LOD | LOQ | LOQ |
| Lamiaceae | *Clerodendrum* | *welwitschii* | le | S/L | 38.0 | 51.1 |  | LOD |  | LOD |  | LOD | 19.7 | 21.1 | 54.9 | 59.0 | 428.8 | 448.0 |  | LOD |  | LOD |  | LOD |
| Lamiaceae | ***Haumaniastrum*** | ***katangense****^#^* | in | aH | 4,051.5^‡^ | 7,063.3^‡^ |  | LOD |  | LOD |  | LOD |  | LOD | LOD | LOD | 419.8 | 423.7 |  | LOD |  | LOD |  | LOD |
| Loganiaceae | *Strychnos* | *cocculoides** | le | S/T | 109.8 | 628.4 |  | LOD |  | LOD | LOD | 3.9 | LOQ | 6.6 | 33.3 | 74.6 | 219.7 | 1,511.4 | LOD | 3.9 | LOD | LOQ |  | LOD |
| Loganiaceae | *Strychnos* | *dale* | le | L | 823.1 | 826.1 |  | LOD |  | LOD |  | LOD | 11.2 | 13.4 | 83.6 | 89.3 | 2,854.4 | 2,862.9 | 8.4 | 10.0 |  | LOD |  | LOD |
| Loganiaceae | *Strychnos* | *pungens^#^* | le | S/T | 94.0 | 106.9 |  | LOD |  | LOD |  | LOD | 8.4 | 9.0 | 35.8 | 39.4 | 993.7 | 1,034.9 |  | LOD |  | LOD |  | LOD |
| Malvaceae | *Hibiscus* | *acetosella* | le | H/sS | 485.2 | 591.6 |  | LOD |  | LOD |  | LOD | LOQ | LOQ | 526.3 | 567.3 | 64.8 | 74.0 |  | LOD |  | LOD |  | LOD |
| Malvaceae | *Hibiscus* | *nigricaulis* | le | a/baH | 112.5 | 135.1 |  | LOD |  | LOD |  | LOD | LOQ | LOQ | 101.2 | 117.5 | 705.1 | 849.3 | 5.6 | 5.9 |  | LOD |  | LOD |
| Malvaceae | ***Hibiscus*** | ***rhodanthus*****^#^* | le | pH | 995.6^‡^ | 6,066.2^‡^ |  | LOD |  | LOD |  | LOD | LOD | 18.5 | 231.9 | 2,569.3 | 178.9 | 609.5 |  | LOD |  | LOD |  | LOD |
| Malvaceae | *Hibiscus* | *sabdariffa* | le | a/baH | 355.6 | 457.1 |  | LOD |  | LOD |  | LOD | LOD | LOQ | 397.5 | 457.1 | 228.6 | 230.1 |  | LOD |  | LOD |  | LOD |
| Malvaceae | *Sida* | *acuta*^#^* | le | a/pH/sS | 101.5 | 613.1 |  | LOD |  | LOD |  | LOD | LOQ | 16.1 | 133.0 | 463.2 | 88.6 | 944.7 | LOD | 4.9 |  | LOD | LOD | LOQ |
| Malvaceae | *Sida* | *cordifolia* | in | aH/sS | 145.1 | 282.4 |  | LOD |  | LOD |  | LOD | LOQ | 7.8 | 117.6 | 189.8 | 352.9 | 445.7 |  | LOD |  | LOD | 102.0 | 110.3 |
| Malvaceae | *Sida* | *cordifolia^#^* | le, in | aH/sS | 104.9 | 115.1 |  | LOD |  | LOD |  | LOD | 6.4 | 7.5 | 85.0 | 91.7 | 407.2 | 419.8 | 2.1 | 2.5 |  | LOD | LOQ | LOQ |
| Malvaceae | *Sida* | *rhombifolia* | le | pH/sS | 119.6 | 142.3 |  | LOD |  | LOD |  | LOD | LOQ | LOQ | 91.1 | 98.1 | 34.2 | 34.3 |  | LOD |  | LOD |  | LOD |
| Malvaceae | ***Waltheria*** | ***indica*****^#^* | le | (s)S | 369.3 | 1,011.1^‡^ |  | LOD |  | LOD |  | LOD | LOQ | LOQ | 375.0 | 759.1 | 85.2 | 236.8 | LOD | 5.7 |  | LOD |  | LOD |
| Moraceae | *Ficus* | *asperifolia** | le | S/T | 50.5 | 138.2 |  | LOD |  | LOD |  | LOD | 6.1 | 10.3 | 58.6 | 211.1 | 68.7 | 267.5 | LOD | 3.3 |  | LOD | LOD | LOQ |
| Moraceae | *Ficus* | *exasperata** | le | T | LOQ | 112.5 |  | LOD |  | LOD |  | LOD | LOQ | 5.5 | 61.8 | 285.2 | 31.1 | 211.2 |  | LOD |  | LOD | LOD | LOQ |
| Moraceae | *Ficus* | *thonningii* | le | T | 111.1 | 149.4 |  | LOD |  | LOD |  | LOD | 4.4 | 4.9 | 93.8 | 125.2 | 61.5 | 64.2 |  | LOD |  | LOD |  | LOD |
| Moraceae | *Ficus* | *thonningii* | fr | T | 13.1 | 17.2 |  | LOD |  | LOD |  | LOD | 8.6 | 9.1 | 61.4 | 64.0 | 28.7 | 32.0 |  | LOD |  | LOD | LOQ | LOQ |
| Moraceae | *Ficus* | *vogeliana* | le | T | 490.2 | 558.9 |  | LOD |  | LOD |  | LOD | 7.6 | 8.2 | 318.6 | 357.4 | 285.9 | 300.4 |  | LOD |  | LOD | LOQ | LOQ |
| Ochnaceae | *Ochna* | *afzelii** | le | S/T | LOQ | 201.6 |  | LOD |  | LOD |  | LOD | LOQ | 18.5 | LOQ | 171.8 | 274.7 | 1,870.8 | LOD | 14.8 |  | LOD | LOD | LOQ |
| Ochnaceae | *Ochna* | *hiernii* | le | S | 77.4 | 114.9 |  | LOD |  | LOD |  | LOD | 9.6 | 9.7 | 96.7 | 124.5 | 1,847.2 | 2,394.6 | 9.6 | 9.7 |  | LOD |  | LOD |
| Ochnaceae | *Ochna* | *multiflora* | le | T | LOQ | LOQ |  | LOD |  | LOD |  | LOD | LOQ | LOQ | LOQ | LOQ | 751.7 | 771.1 | 8.1 | 8.4 |  | LOD |  | LOD |
| Ochnaceae | *Ochna* | *pulchra** | le | S/T | LOQ | 237.7 |  | LOD |  | LOD |  | LOD | LOQ | 7.0 | LOQ | 132.3 | 196.4 | 440.0 | LOD | 4.5 |  | LOD | LOD | LOQ |
| Ochnaceae | *Ochna* | *pulchra* | in, fr | S/T | LOD | LOQ |  | LOD |  | LOD |  | LOD | 9.0 | 11.4 | 24.0 | 34.1 | 43.6 | 56.9 | 1.5 | 1.6 |  | LOD | LOQ | LOQ |
| Ochnaceae | *Ochna* | *pygmaea*^#^* | le | (s)S | 27.0 | 239.3 |  | LOD |  | LOD |  | LOD | 3.5 | 8.3 | 58.9 | 98.9 | 284.5 | 1,246.6 | 2.6 | 5.4 |  | LOD | LOD | LOQ |
| Ochnaceae | *Rabdophyllum* | *welwitschii** | le | S/T | 57.3 | 149.7 |  | LOD | LOD | 5.7 |  | LOD | LOQ | 6.6 | 71.6 | 140.3 | 1,198.6 | 2,059.8 | 2.9 | 10.3 |  | LOD | LOQ | 62.4 |
| Olacaceae | *Olax* | *gambecola** | le | S | LOQ | 208.5 |  | LOD |  | LOD |  | LOD | 12.7 | 37.2 | 45.4 | 174.1 | 654.6 | 2,072.9 | 4.2 | 8.3 |  | LOD | LOQ | 116.8 |
| Olacaceae | *Olax* | *gambecola* | fr | S |  | LOD |  | LOD |  | LOD |  | LOD | 7.5 | 10.1 | 33.6 | 33.9 | 60.2 | 70.5 | 3.4 | 3.8 |  | LOD | LOQ | LOQ |
| Orobanchaceae | *Buchnera* | *henriquesii* | le | aH | 83.7 | 127.5 |  | LOD |  | LOD |  | LOD | LOQ | 15.2 | 60.9 | 78.4 | 175.0 | 215.7 | 7.6 | 9.8 |  | LOD |  | LOD |
| Orobanchaceae | *Buchnera* | *lippioides* | le | a/pH | 76.4 | 87.8 |  | LOD |  | LOD |  | LOD | LOQ | 10.3 | 105.8 | 108.5 | 41.1 | 51.7 |  | LOD |  | LOD |  | LOD |
| Orobanchaceae | *Buchnera* | *lippioides* | in | a/pH | LOD | LOQ |  | LOD |  | LOD |  | LOD | LOQ | LOQ | LOQ | LOQ | 16.6 | 21.4 |  | LOD |  | LOD | LOD | LOQ |
| Phyllanthaceae | *Antidesma* | *laciniatum* | le | T | 61.9 | 76.3 |  | LOD |  | LOD |  | LOD | 4.8 | 4.9 | 78.6 | 91.1 | 1,948.5 | 1,976.9 |  | LOD |  | LOD | LOD | LOQ |
| Phyllanthaceae | *Antidesma* | *laciniatum* | fr | T |  | LOD |  | LOD |  | LOD |  | LOD | 4.7 | 6.7 | 29.2 | 30.8 | 271.4 | 277.1 | 2.2 | 2.4 |  | LOD | LOQ | LOQ |
| Phyllanthaceae | *Antidesma* | *venosum** | le | S/T | 89.0 | 142.8 |  | LOD | LOD | 1.4 |  | LOD | 4.0 | 24.3 | 96.7 | 150.9 | 663.8 | 3,956.4^‡^ | LOD | 1.4 | LOD | LOQ | LOQ | LOQ |
| Phyllanthaceae | *Antidesma* | *venosum* | fr | S/T | 16.5 | 23.2 |  | LOD |  | LOD |  | LOD | 6.6 | 7.3 | 23.8 | 29.9 | 212.4 | 240.5 | 1.7 | 1.8 |  | LOD | LOQ | LOQ |
| Phyllanthaceae | *Antidesma* | *vogelianum* | le | S/T | 112.9 | 131.4 |  | LOD |  | LOD |  | LOD | 4.2 | 5.4 | 95.0 | 110.5 | 1,038.4 | 1,173.8 | 1.8 | 2.1 |  | LOD | LOQ | LOQ |
| Phyllanthaceae | *Phyllanthus* | *nummulariifolius* | le | (s)S |  | LOD |  | LOD |  | LOD |  | LOD | LOQ | LOQ | LOQ | LOQ | 147.9 | 290.0 | LOD | 9.9 |  | LOD |  | LOD |
| Phyllanthaceae | *Phyllanthus* | *ovalifolius** | le | S/T | LOD | LOQ |  | LOD | LOD | 6.9 |  | LOD | LOQ | 9.7 | LOQ | 77.3 | 103.8 | 185.5 |  | LOD |  | LOD | LOD | LOQ |
| Phyllanthaceae | *Phyllanthus* | *physocarpus* | le | S | LOQ | LOQ |  | LOD |  | LOD |  | LOD | LOQ | LOQ | LOQ | 41.8 | 159.8 | 199.2 |  | LOD |  | LOD |  | LOD |
| Polygalaceae | *Polygala* | *acicularis** | le | (s)S | LOQ | 821.9 |  | LOD |  | LOD |  | LOD | LOQ | LOQ | 78.7 | 469.7 | 202.2 | 368.8 |  | LOD |  | LOD |  | LOD |
| Polygalaceae | *Polygala* | *albida* | le | aH | 242.7 | 363.6 |  | LOD |  | LOD |  | LOD |  | LOD | 145.6 | 236.4 | 72.7 | 97.1 |  | LOD |  | LOD |  | LOD |
| Polygalaceae | *Polygala* | *gomesiana* | le | pH | 117.6 | 140.7 |  | LOD |  | LOD |  | LOD | LOQ | LOQ | 132.4 | 145.4 | 701.0 | 755.2 |  | LOD |  | LOD |  | LOD |
| Polygalaceae | *Polygala* | *macrostigma* | le | aH | 189.2 | 196.2 |  | LOD |  | LOD |  | LOD | LOQ | LOQ | 183.5 | 190.2 | 499.4 | 550.5 |  | LOD |  | LOD |  | LOD |
| Pteridaceae | ***Pityrogramma*** | ***calomelanos*****^#^* | le | H | LOD | 2,128.5^‡^ |  | LOD |  | LOD | 2.3 | 2.4 | LOQ | 10.0 | LOQ | 913.3 | 22.6 | 105.4 | LOQ | 7.1 | LOQ | LOQ |  | LOD |
| Pteridaceae | *Pteris* | *friesii* | le | H | 273.4 | 380.0 |  | LOD |  | LOD |  | LOD | LOQ | LOQ | 279.9 | 355.9 | 114.6 | 130.2 |  | LOD |  | LOD | LOD | LOQ |
| Rubiaceae | *Psychotria* | *globiceps** | le | S | 183.3 | 247.0 |  | LOD |  | LOD |  | LOD | 4.6 | 7.0 | 76.0 | 139.8 | 115.8 | 409.9 |  | LOD |  | LOD | LOD | LOQ |
| Rubiaceae | *Psychotria* | *peduncularis** | le | (s)S | 178.6 | 505.6 |  | LOD |  | LOD |  | LOD | LOQ | 21.3 | 80.0 | 198.2 | 2,650.5^‡^ | 4,696.4^‡^ | LOD | 7.3 |  | LOD | LOD | LOQ |
| Rubiaceae | ***Spermacoce*** | ***dibrachiata**** | le | aH/sS | 2,534.8^‡^ | 2,977.6^‡^ |  | LOD |  | LOD |  | LOD | LOD | LOQ | 147.8 | 506.7 | 283.0 | 295.6 |  | LOD |  | LOD |  | LOD |
| Rubiaceae | ***Spermacoce*** | ***pusilla*** | le | aH | 1,484.2^‡^ | 1,860.2^‡^ |  | LOD |  | LOD |  | LOD |  | LOD | 441.8 | 649.4 | 160.6 | 194.8 |  | LOD |  | LOD |  | LOD |
| Rubiaceae | ***Spermacoce*** | ***quadrisulcata*** | le | aH | 1,315.9^‡^ | 2,176.9^‡^ |  | LOD |  | LOD |  | LOD | LOQ | 11.0 | 53.4 | 60.5 | 53.4 | 66.0 |  | LOD |  | LOD | LOQ | LOQ |
| Rubiaceae | ***Spermacoce*** | ***senensis**** | le | aH | 1,306.5 | 3,316.4^‡^ |  | LOD |  | LOD |  | LOD | LOD | LOQ | LOQ | 1,996.3 | 80.2 | 301.4 |  | LOD |  | LOD |  | LOD |
| Solanaceae | *Solanum* | *aculeastrum** | le | S/T | 91.9 | 487.7 |  | LOD |  | LOD |  | LOD | 12.9 | 41.9 | 107.8 | 440.7 | 31.7 | 275.7 | LOD | 2.2 |  | LOD | LOQ | LOQ |
| Solanaceae | *Solanum* | *aethiopicum* | le | (s)S | 113.6 | 125.3 |  | LOD |  | LOD |  | LOD | LOQ | LOQ | 178.6 | 186.1 | 34.5 | 37.9 |  | LOD |  | LOD | LOQ | LOQ |
| Solanaceae | *Solanum* | *americanum* | le | a/pH | 539.4 | 647.9 |  | LOD |  | LOD |  | LOD | 11.7 | 12.8 | 440.9 | 484.0 | 94.2 | 105.4 |  | LOD |  | LOD | LOQ | LOQ |
| Solanaceae | *Solanum* | *anguivi* | le | (s)S | LOQ | LOQ |  | LOD |  | LOD |  | LOD | LOQ | LOQ | LOQ | LOQ | 33.3 | 58.1 |  | LOD |  | LOD |  | LOD |
| Solanaceae | *Solanum* | *macrocarpon* | le | S | 234.2 | 250.8 |  | LOD |  | LOD |  | LOD | 16.2 | 16.2 | 210.0 | 218.4 | 364.1 | 484.7 |  | LOD |  | LOD |  | LOD |
| Solanaceae | ***Solanum*** | ***mauritianum**** | le | S/T | 35.1 | 2,998.6^‡^ |  | LOD |  | LOD | LOD | 3.4 | LOQ | 10.3 | 64.4 | 3,196.1 | 19.7 | 102.1 | LOD | 3.4 |  | LOQ | LOD | LOQ |
| Solanaceae | *Solanum* | *mauritianum* | in | S/T | 82.1 | 104.3 |  | LOD |  | LOD |  | LOD | 10.0 | 10.6 | 104.2 | 119.3 | 44.1 | 51.1 | 2.0 | 2.1 |  | LOD | LOQ | LOQ |
| Solanaceae | *Solanum* | *terminale* | le | L | 111.5 | 119.6 |  | LOD |  | LOD |  | LOD | 9.0 | 9.7 | 90.5 | 120.6 | 1,515.8 | 1,567.2 |  | LOD |  | LOD | LOQ | 60.3 |
| Solanaceae | *Solanum* | *terminale* | in | L | 21.1 | 24.2 |  | LOD |  | LOD |  | LOD | 10.5 | 12.1 | 35.1 | 40.4 | 73.7 | 80.8 |  | LOD |  | LOD |  | LOD |
| Urticaceae | *Laportea* | *aestuans** | le | aH | LOD | 62.2 |  | LOD |  | LOD |  | LOD | LOQ | 9.3 | 59.0 | 95.7 | 23.6 | 38.3 |  | LOD |  | LOD | LOD | LOQ |
| Urticaceae | *Laportea* | *aestuans* | fr | aH | 12.8 | 19.8 |  | LOD |  | LOD |  | LOD | 9.9 | 10.2 | 69.1 | 135.0 | 30.7 | 36.2 |  | LOD |  | LOD | LOQ | LOQ |
| Urticaceae | *Laportea* | *mooreana** | le | aH/sS | LOQ | 57.7 |  | LOD |  | LOD |  | LOD | LOQ | 7.5 | 105.3 | 184.8 | 148.3 | 163.0 |  | LOD |  | LOD | LOD | LOQ |
| Urticaceae | *Laportea* | *ovalifolia** | le | pH | 20.9 | 167.0 |  | LOD |  | LOD |  | LOD | LOD | 9.9 | 75.9 | 187.9 | 83.5 | 464.9 |  | LOD |  | LOD | LOD | LOQ |
| Verbenaceae | *Lippia* | *abyssinica* | le | (s)S | 89.8 | 102.0 |  | LOD |  | LOD |  | LOD | 5.8 | 6.0 | 89.8 | 93.2 | 35.9 | 37.9 |  | LOD |  | LOD | LOQ | LOQ |
| Verbenaceae | *Lippia* | *multiflora* | in | (s)S | 900.7 | 936.5 |  | LOD |  | LOD |  | LOD | 14.2 | 15.5 | 874.6 | 936.2 | 31.0 | 35.5 |  | LOD |  | LOD | LOD | LOQ |
| Violaceae | *Rinorea* | *brachypetala** | le | S/T | 62.9 | 200.9 |  | LOD |  | LOD |  | LOD | LOQ | 21.2 | 67.7 | 110.5 | 62.9 | 719.3 | 3.3 | 4.8 |  | LOD | LOD | 74.0 |
| Violaceae | *Rinorea* | *welwitschii** | le | S/T | 45.5 | 195.0 |  | LOD |  | LOD | LOD | 3.0 | 8.6 | 10.3 | 75.9 | 130.0 | 111.6 | 123.5 |  | LOD |  | LOD | LOD | LOQ |
